# Amplitude-dependency of response of SI cortex to flutter stimulation

**DOI:** 10.1186/1471-2202-6-43

**Published:** 2005-06-21

**Authors:** Stephen B Simons, Vinay Tannan, Joannellyn Chiu, Oleg V Favorov, Barry L Whitsel, Mark Tommerdahl

**Affiliations:** 1 Departments of Biomedical Engineering, University of North Carolina at Chapel Hill, Chapel Hill, North Carolina 27599, USA; 2 Cellular and Molecular Physiology, University of North Carolina at Chapel Hill, Chapel Hill, North Carolina 27599, USA

## Abstract

**Background:**

It is established that increasing the amplitude of a flutter stimulus increases its perceived intensity. Although many studies have examined this phenomenon with regard to the responding afferent population, the way in which the intensity of a stimulus is coded in primary somatosensory cortex (SI) remains unclear.

**Results:**

Optical intrinsic signal (OIS) imaging was used to study the evoked responses in SI of anesthetized squirrel monkeys by 25 Hz sinusoidal vertical skin displacement stimulation. Stimuli were 10 sec duration with a 50 sec inter-stimulus interval. Stimulus amplitude ranged from 50 to 400 microns and different amplitudes were interleaved. Control levels of activity were measured in the absence of stimulation, and used to compare with activation levels evoked by the different stimulus amplitudes. Stimulation of a discrete skin site on the forelimb evoked a prominent increase in absorbance within the forelimb representational region in cytoarchitectonic areas 3b and 1 of the contralateral hemisphere. An increase in stimulus amplitude led to a proportional increase in the magnitude of the absorbance increase in this region of areas 3b and 1 while surrounding cortex underwent a decrease in absorbance. Correlation maps revealed that as stimulus amplitude is increased, the spatial extent of the activated region in SI remains relatively constant, and the activity within this region increases progressively. Additionally, as stimulus amplitude is increased to suprathreshold levels, activity in the surround of the activated SI territory decreases, suggesting an increase in inhibition of neuronal activity within these regions.

**Conclusion:**

Increasing the amplitude of a flutter stimulus leads to a proportional increase in absorbance within the forelimb representational region of SI. This most likely reflects an increase in the firing rate of neurons in this region of SI. The relatively constant spatial extent of this stimulus-evoked increase in absorbance suggests that an increase in the amplitude of a 25 Hz skin stimulus does not evoke a larger area of SI neuronal activation due to an amplitude-dependent lateral inhibitory effect that spatially funnels the responding SI neuronal population.

## Background

The way in which stimulus intensity is represented in primary somatosensory (SI) cortex has remained an intriguing question in the study of the cortical correlates of perception. Although there have been numerous studies of the SI response to changes in stimulus intensity, few have focused on the response at the population level of analysis. Thus, a dearth of information about the global SI response to changes in stimulus intensity exists – current knowledge of the subject depends almost exclusively on reconstruction of predictions of the SI neuronal population response from afferent recordings [1-5] and single unit cortical recordings [6,7].

A number of studies have examined the global SI response using imaging techniques such as fMRI (functional Magnetic Resonance Imaging) [8-10] and MEG (MagnetoEncephaloGraphy)[11,12]. In general, results from these studies indicate that increases in stimulus intensity are accompanied by increases in the intensity of the evoked signal as well as increases in the activated volume of cortex. As a result these studies predicted that amplitude might be coded not only by the average firing rates of individual SI neurons, but also by the total aggregate of responding neurons. However, although each of these imaging techniques provide measures which are indirectly related to neuronal activity, their resolution is limited in two important ways. First, it is difficult to determine the nature of the neuronal activity being imaged (whether or not it is excitatory or inhibitory); and second, both fMRI and MEG studies have limited spatial resolution, which is typically on the order of ~1 mm^2 ^[8,11].

Recently, Chen and colleagues used the optical intrinsic signal (OIS) to demonstrate that a proportionally greater (larger magnitude) response is evoked in SI of squirrel monkeys as the amplitude (as measured by force) of a skin stimulus is increased[13]. However, the primary focus of their report was that the response to simultaneous stimulation of multiple adjacent sites on the skin produced a smaller, more intense region of SI activation than would be normally predicted by summation of the two, and their findings did not detail the effects of amplitude on the dynamics of the response to a flutter stimulus at a single site on the skin. In this report, we extend the aforementioned work by imaging the OIS evoked in SI cortex of squirrel monkeys by a range of amplitudes of skin flutter stimulation. The results suggest that increasing the amplitude of a skin flutter stimulus evokes a proportionally larger absorbance increase in SI that remains confined to the same SI territory. In addition, it was found that increasing the amplitude of flutter evokes a large decrease in absorbance in the territory that borders the activated region of SI. Neurons in the SI region that demonstrate decreased absorbance in response to flutter stimulation are proposed to undergo stimulus-evoked inhibition and to contribute importantly to the SI processing of high-amplitude skin flutter stimuli.

## Results

**Figure 1:**
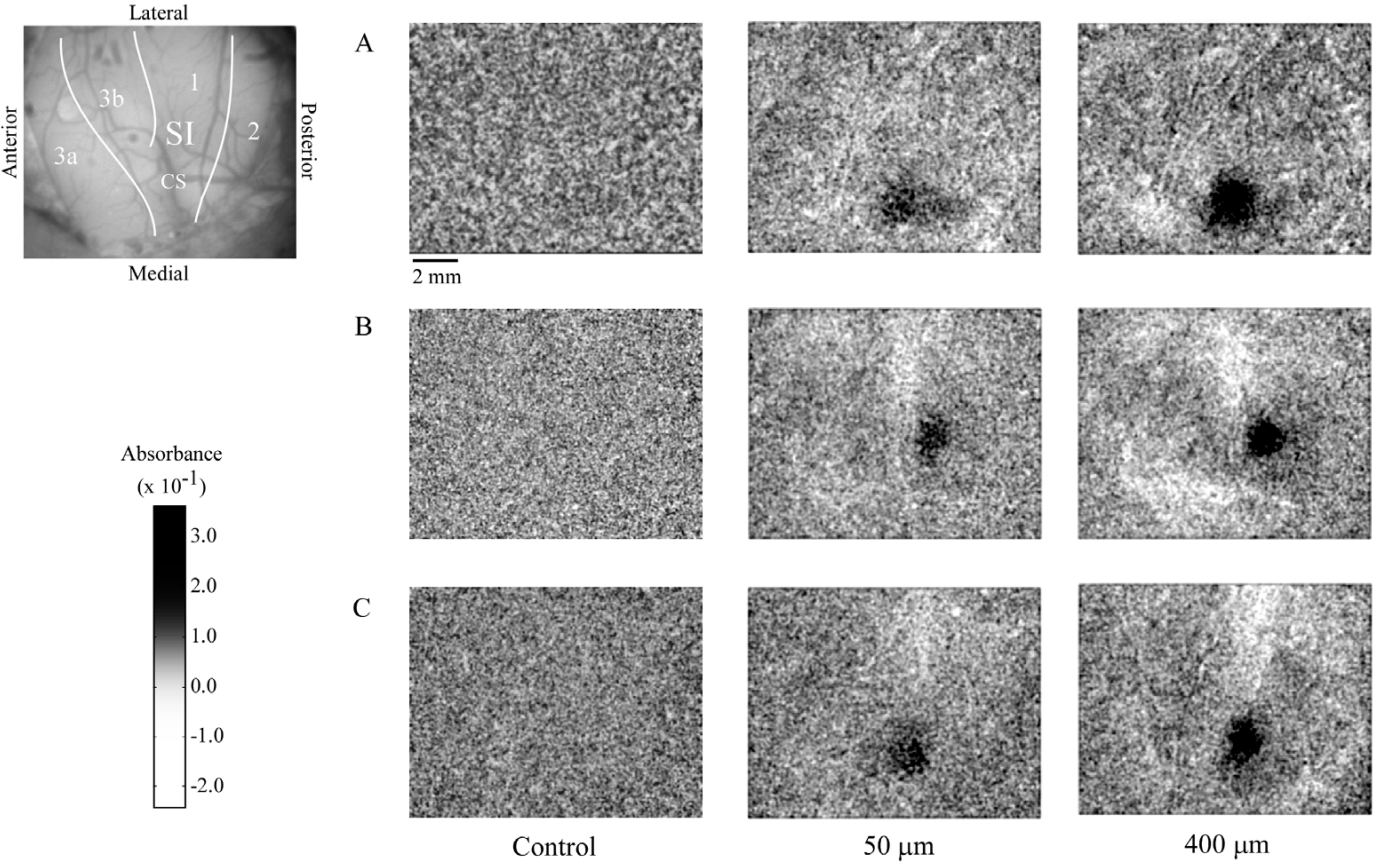
OIS response to low vs. high amplitude stimulation. OIS images taken from three subjects (A, B, C). All images are anatomically oriented as shown in the top left image. Images were obtained by averaging across 10 experimental trials and then summing frames taken from the time of stimulus onset to 5 seconds after stimulus offset to better show regions of high absorbance indicated by dark pixels. The left column shows responses acquired in the absence of stimulation (control), while the middle and right columns show the stimulus-evoked response to 50 and 400 μm respectively in the same subjects.

Figure 1(A–C) illustrates typical examples of the OIS response in SI of three different subjects in the absence of stimulation (control), and during low (50 μm) versus high (400 μm) amplitude stimulation. Each image shown in Figure 1 represents the sum of frames taken from the time of stimulus onset to 5 seconds after stimulus offset (frames 1–16). Areas of high absorbance are indicated by dark patches within each image; regions of high absorbance in each case correspond to the SI locus that represents the stimulated site on the skin. SI in each experiment underwent a larger increase in absorbance within the region of interest (ROI) in response to the 400 μm stimulus than evoked in the same region by the 50 μm stimulus. Moreover, in each subject the increase in absorbance appears more evenly distributed and less diffuse throughout the ROI under the 400 μm condition.

**Figure 2:**
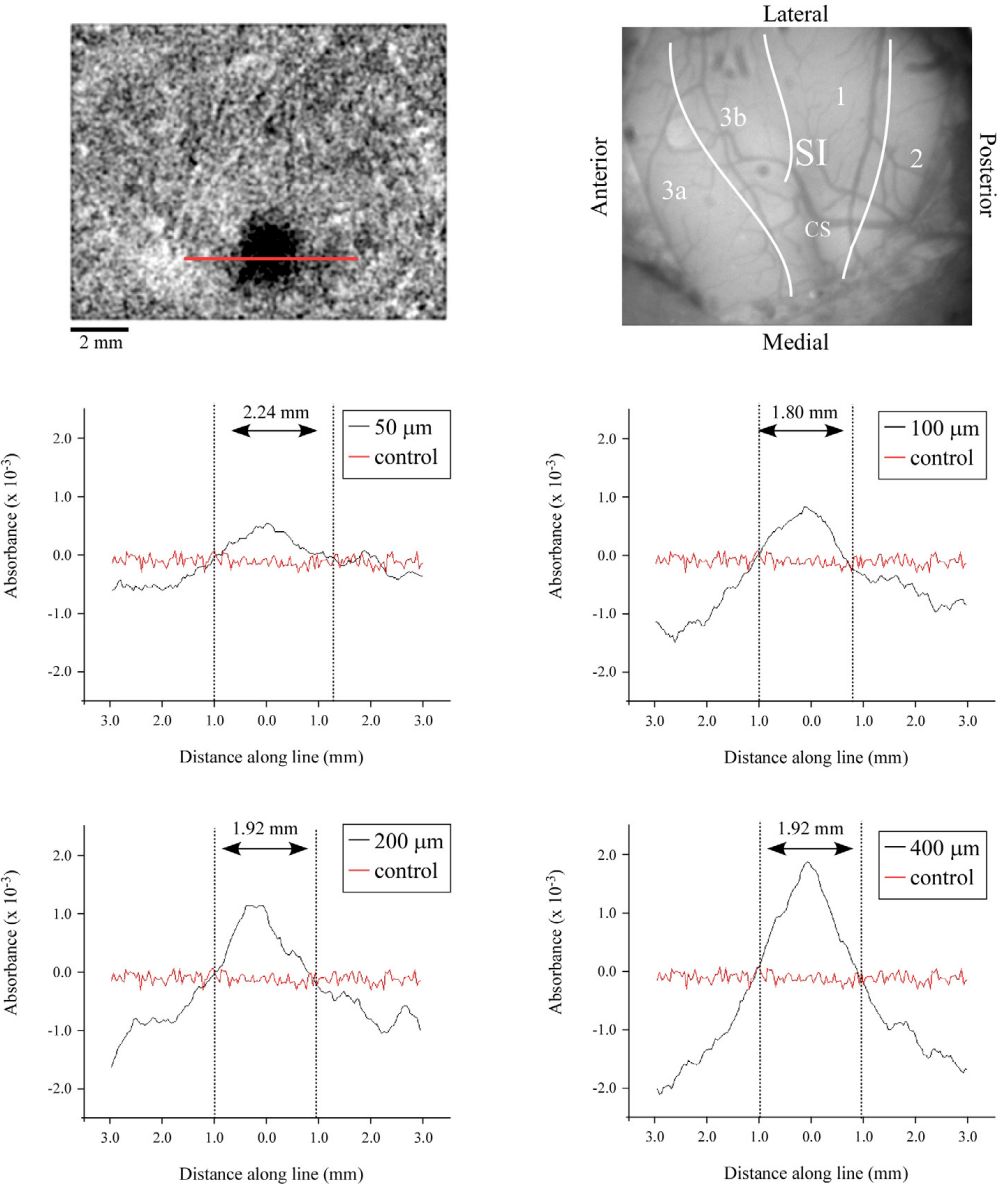
Spatial histograms of activity at different amplitudes. Absorbances were measured at each amplitude along the red line shown in the OIS image at top left. Each plotted value represents an average of pixels spanning 100 μm above and below the line and a distance of 40 μm along the line (bin size was 40 × 200 μm). The control condition is plotted on each graph to indicate "background" levels absorbance. Dashed lines on plots indicate where stimulus-evoked activity crosses background absorbance levels (indicating the boundaries of above background absorbance). Histograms indicate no significant change in cortical territory displaying above background absorbance with respect to stimulus amplitude. Higher amplitude stimulation produces regions of below-background absorbance directly outside of the regions of above-background absorbance.

To identify the boundaries of the regions of increased absorbance spatial histograms were constructed. Figure 2 illustrates the average results obtained from all experiments (n = 5) as well as the methodology used to evaluate the spatial extent of the stimulus-evoked activation. In each experiment, the image was segmented along a line 6 mm long and roughly centered on the area of increased absorbance, as shown in the top panel. Pixels along the line were binned (bin size 40 × 200 μm) and absorbance values averaged and plotted as a function of distance along the line. The plots demonstrate that at all amplitudes of stimulation, the spatial extent of the region of above-background absorbance (ie. absorbance values larger than control) is similar and at every stimulus amplitude occupies a circular-shaped territory in SI between 1.8 – 2.24 mm in diameter. The ROI (to be used for further analysis) was therefore defined as the region displaying above background levels of absorbance within the activated region of SI.

**Figure 3:**
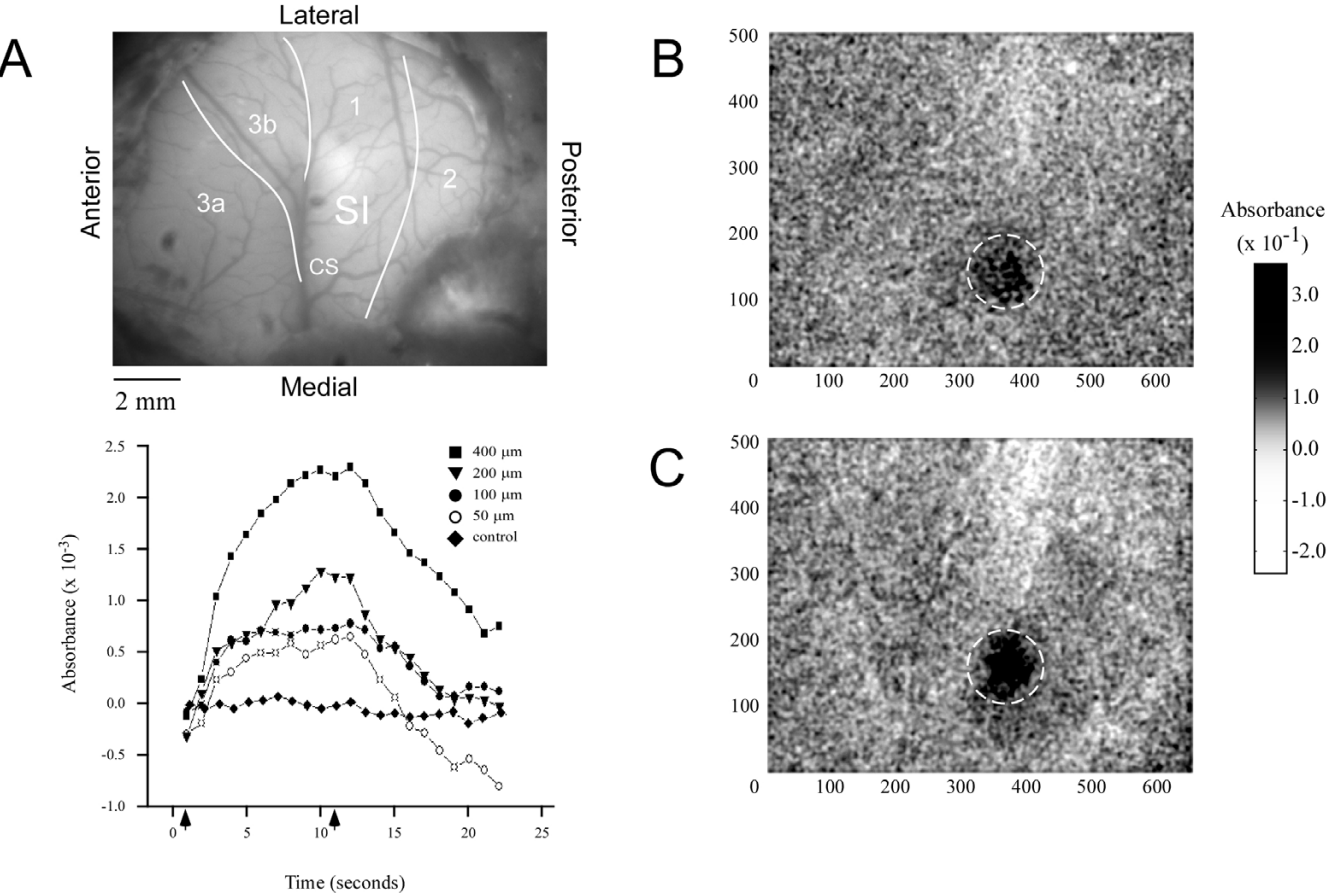
Absorbance time course and anatomical registration in SI. *A*) Green filter image of SI cortex in vivo, used for anatomical registration of OIS images. *B&C*) Resulting OIS image obtained from averaging across 10 experimental trials and then summing frames taken from the time of stimulus onset to 5 seconds after stimulus offset: *B*) at stimulus amplitude 50 μm, *C*) at 400 μm. Dashed circles enclose the ROI within SI. *Bottom left*) Plot of the averaged absorbance. Arrows indicate time of stimulus onset (1s) and stimulus offset (11s). Absorbance values within the enclosed ROI were averaged and plotted at each amplitude as a function of time.

Figure 3 demonstrates (for one exemplary subject) the method used to evaluate the time course of stimulus-evoked SI absorbance. Panel A shows a green filter image of the cortical surface, which highlights the vasculature. Panels B&C are the OIS responses (dark regions) evoked by the low (50 μm) and high (400 μm) stimulus amplitudes respectively. The ROI is the circular territory enclosed by the dashed white lines. Absorbance values within the ROI were averaged for each amplitude of stimulation and plotted as a function of time. The time course of the absorbance values measured between 1 and 22 sec after stimulus onset is plotted for each of the stimulus conditions, and in the absence of stimulation ("control"). Arrows along x-axis of plots at bottom left of Figure 3 indicate stimulus onset (1 sec) and stimulus offset (11 sec), and reveal how absorbance increased with increasing amplitude of stimulation. For each stimulus amplitude absorbance is maximal near to stimulus offset.

**Figure 4:**
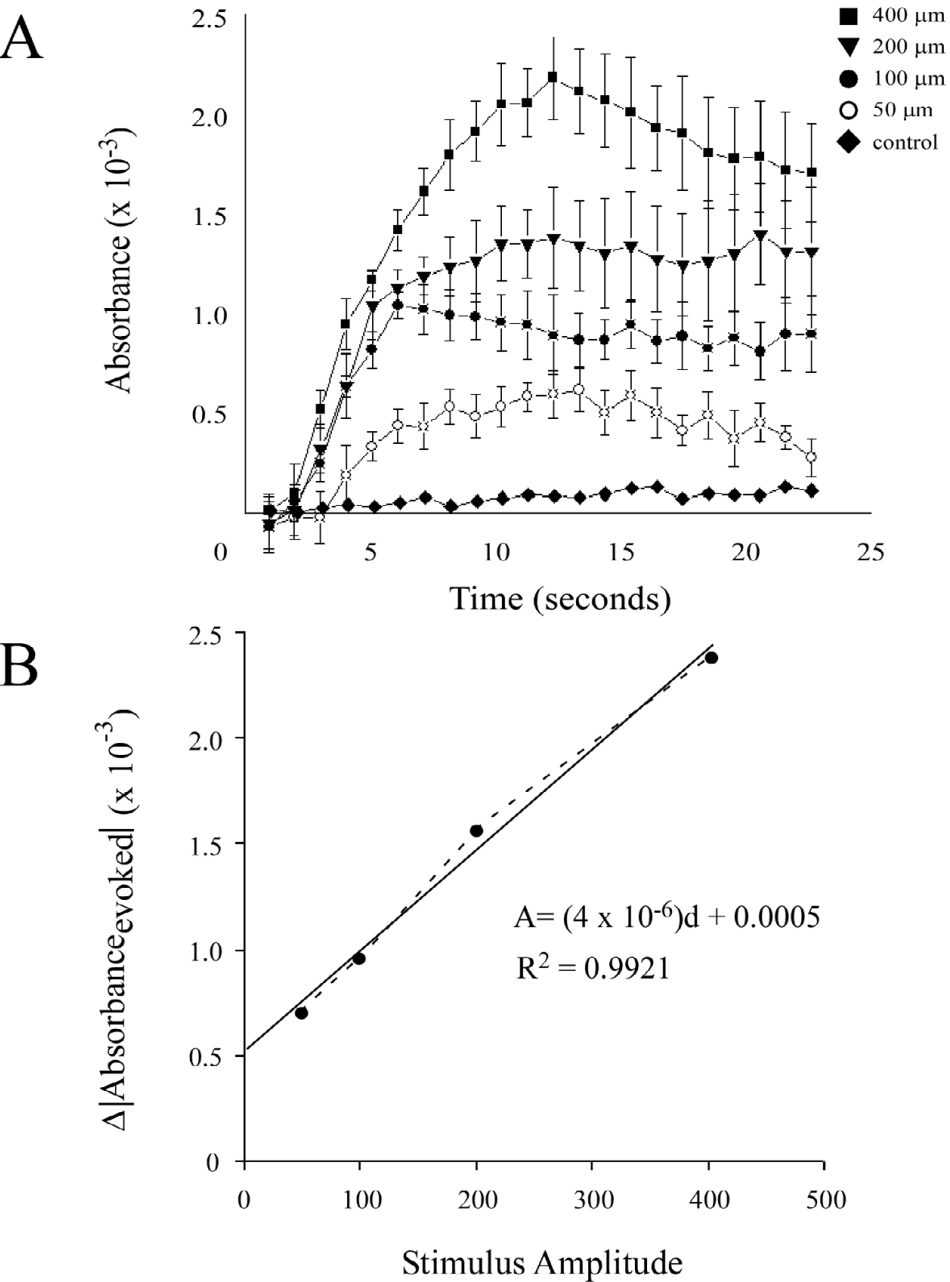
*A*) Plots of absorbance and standard deviation averaged across all experiments (n = 5). All data was normalized prior to being averaged. Plotted absorbances were measured within the ROI which was defined as all pixels within a 1 mm radius of the center of activation (as defined from spatial histogram analysis). Controls in which no stimulus was administered are plotted simultaneously for comparison to test conditions. *B*) Plot of ΔAbsorbance_evoked _which was defined as the change in absorbance measured from frame 1 (prior to stimulus onset) to frame 11 (point of stimulus offset). The plot is fit with a linear function (solid line) described by the equation A = (4 × 10^-6^)d + 0.0005 where A is absorbance and d is stimulus amplitude (displacement). Coefficient of determination for the linear regression (R^2^) is shown below.

The analysis approach illustrated in Figure 3 was performed on all experiments (n = 5) and the resulting absorbance plots were averaged (Figure 4). Similar to Figure 3, the plots in Figure 4 demonstrate that absorbance increases with increasing stimulus amplitude. To quantify this relationship a measure of ΔAbsorbance_evoked _was used. ΔAbsorbance_evoked _was defined as the difference between the absorbance measured at 1 sec (prior to stimulus onset) and 11 sec (point of stimulus offset), and is shown in the plot at the bottom of Figure 4. The plot of ΔAbsorbance_evoked _vs. amplitude is well described (coefficient of determination R^2 ^= 0.9921) by the linear function (solid line) ΔAbsorbance_evoked _= (4 × 10^-6^)*d + 0.0005. This type of analysis, however, gives little or no information about the spatial properties of the response.

**Figure 5:**
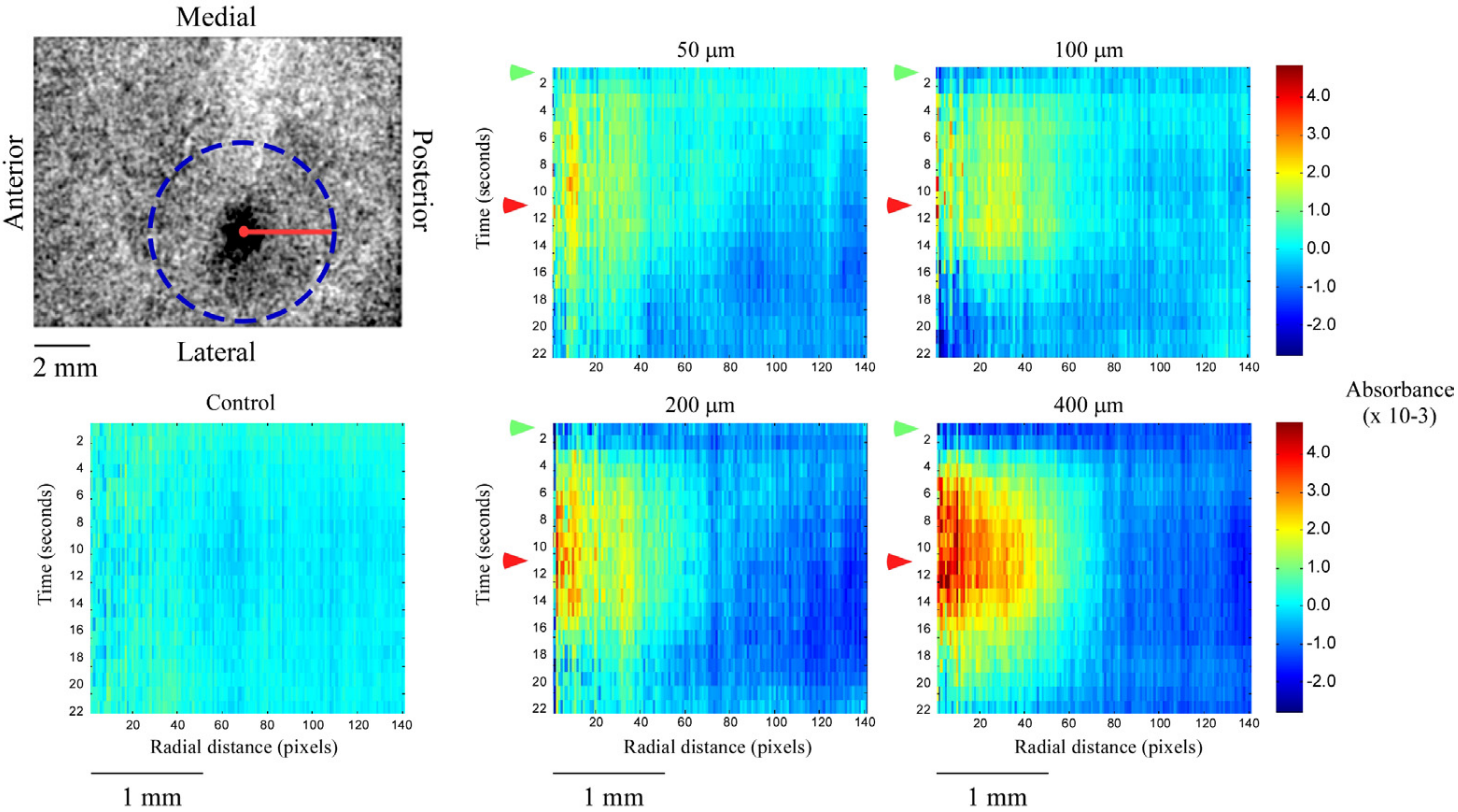
Radial time space plots. Radial histograms were performed on OIS data at all stimulus amplitudes. Radii were measured from the center of activation as demonstrated in the image at top-left. The dashed blue line is the maximum radial distance used in the maps shown. The schematic at bottom left indicates the anatomical orientation of the cortex in the image above. Absorbance values in maps shown represent an average at each radial distance at each frame after stimulus onset. Green arrows indicate stimulus onset (1s) while red arrows indicate stimulus offset (11s). Absorbance values are color coded as per the color bar shown at the right.

Radial histograms were constructed to better visualize the spatiotemporal relationship of the OIS response at different amplitudes. The radial histograms shown in Figure 5 are representative time-space plots at each amplitude. From the ROI center (as determined by spatial histogram analysis), average absorbance values were determined for the pixels within concentric rings located at progressively larger distances from the center at each frame acquired. Absorbance values are color coded (red indicating areas of high absorbance, blue indicating areas of low absorbance) and plotted as a function of time and radial distance from the center of the ROI. Figure 5 demonstrates that the major differences that exist in the SI global responses to different amplitudes of stimulation are differences in the magnitude of absorbance and not the spatial properties of the absorbance pattern (this also is apparent in the spatial histogram analysis of Figure 2). As would be expected based on the absorbance curves shown in Figures 3 &4, higher stimulus amplitudes evoked a more intense and discrete region of increased absorbance than did the lower amplitudes. Interestingly, one of the more robust differences between low- and high-amplitude stimulation, is the magnitude of decreasing absorbance detected in the territory that surrounds the region in which absorbance increases. This response is most pronounced under the 400 μm condition where it can be seen to occur much sooner after stimulus onset at radial distances as small as 1.5 mm from the ROI center. Spatially, the regions of above- and below-background absorbance are nearly the same at each stimulus amplitude, with the above-background portion extending nearly 1 mm away in all directions from the center of activity, whereas the below-background portion of the response comprises a ring beginning at a radial distance of 1.5 mm from the ROI center and extending out beyond the area that was analyzed.

**Figure 6:**
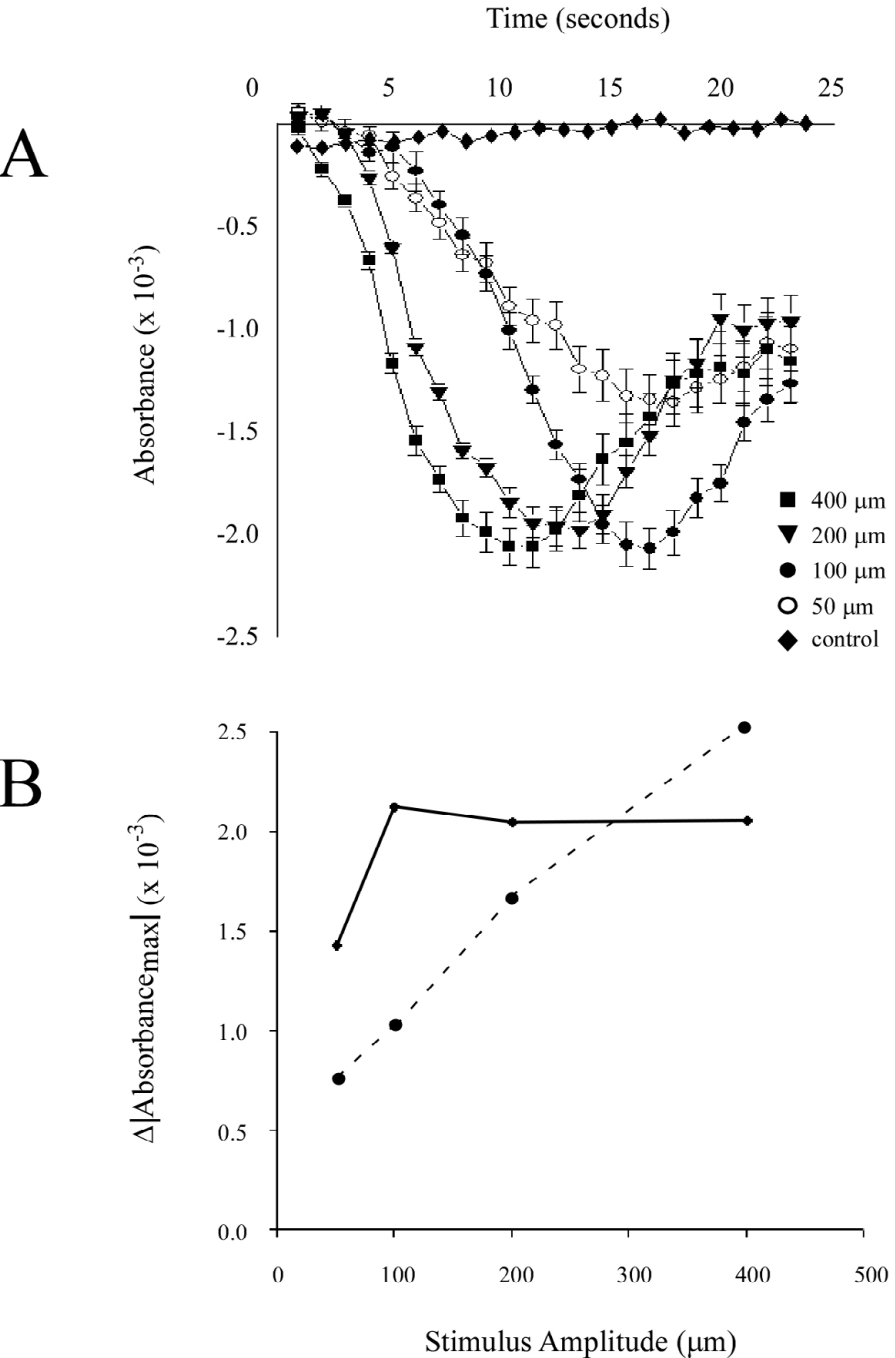
Absorbance trends surrounding the ROI across five experiments. *A*) Plots of absorbance and standard deviation averaged across all experiments (n = 5). Plotted absorbances were measured at radial distances between 1 and 1.5 mm away from the center of the ROI (defined earlier). *B*) Plot of the maximal change in absorbance (solid line) as a function of stimulus amplitude. The linear trend, obtained from averaged absorbance measured within the ROI (dashed line from figure 3) has been simultaneously plotted on the axes to demonstrate the significant differences between the two regions.

Using similar techniques to those we used to analyze above-background activity in the ROI (as in Figures 2 &3), regions outside the designated ROI were examined to determine whether a similar amplitude-dependency could be established for the time courses of the below-background absorbance observed in the surround. Figure 6 shows plots constructed from averaging the absorbance values in pixels lying 1.5 – 2 mm away from the center of the ROI. Data were normalized and then averaged across experiments (n = 5). It is apparent that the time courses at different amplitudes of stimulation are different with respect to the stimulus timing, (compared with above background levels of activity, which all show maximum absorbance at the point of stimulus offset). Accordingly, a different measure was adopted to quantify this relationship: In this case ΔAbsorbance_max _was defined as the difference between the minimum absorbance and the maximum absorbance value obtained at any point during the recording. Interestingly, the relationship between stimulus amplitude and ΔAbsorbance_max _in the surround is not linear (Figure 6b). Instead, each of the higher stimulus amplitudes employed (100–400 μm) evoked a very similar level of below-background absorbance. The sole difference between the different curves (Figure 6a) is the time required to reach the peak of the decrease in absorbance. That is, as amplitude is increased from 100 to 400 μm the point of minimum absorbance was attained earlier in time. This is also apparent in Figure 4.

**Figure 7:**
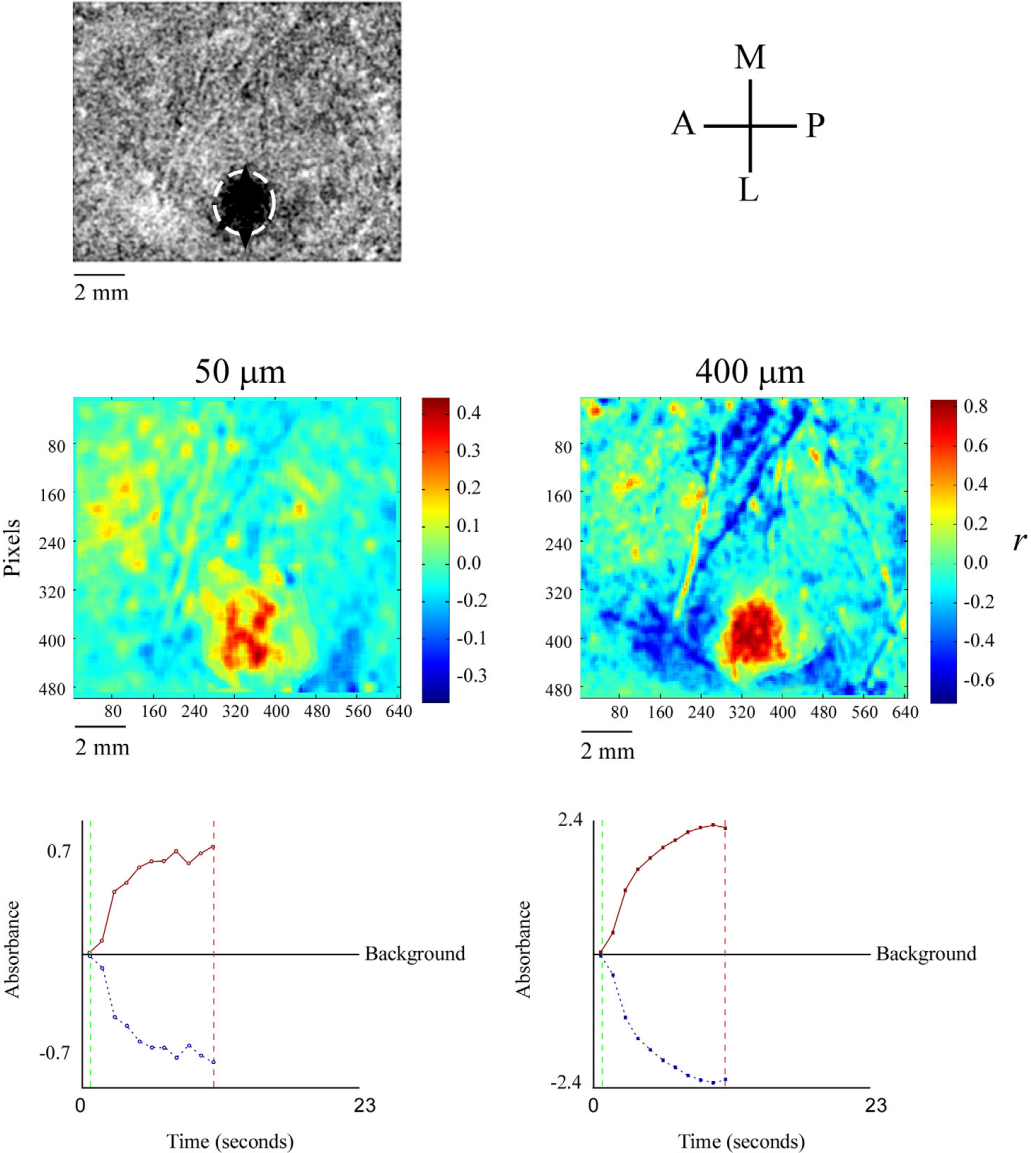
Correlation maps for stimulus amplitudes of 50 (left) and 400 (right) microns. The schematic at top-right indicates the anatomical orientation of the OIS image as well as all maps: A-anterior, M-medial, P-posterior, L-lateral. Color bars show coefficient of determination values for each map. The correlated signal, obtained from averaging of absorbance values within the ROI (encircled in the image at top-left) at each frame, is shown under each map (time course in solid red). Only the portion of the signal enclosed within the dashed lines (corresponding to the stimulus duration, green-on, red-off) is correlated. r values describe the degree of similarity between a given pixel's time course and the corrselated signal. Negative r values indicate that absorbance decreases to below-background levels. Thus, the signal in solid red represents the time course that would be observed in a pixel with an r value of +1 and the dotted blue line indicates the time course that would be observed in a pixel with an r value of -1.

Correlation maps were constructed to further characterize the spatial properties of the SI response to 25 Hz flutter. A correlation map compares every pixel in the image with the signal referenced from the ROI, and assigns a correlation coefficient (*r*) to the location of the pixel being compared. This gives a fairly good approximation of the signal at all locations in the image. Since there is no significant difference in the spatial properties between stimuli at intermediate amplitudes (as demonstrated by radial histograms) only the 50 and 400 μm amplitudes will be compared with this technique. Figure 7 shows correlation maps of the OIS responses to stimulus amplitudes of 50 and 400 μm (Top panels). The bottom panels of the figure show the input signal (solid dark red line) used for correlation of each pixel in the map, and the negative (opposite) of the input signal (dotted blue line). A coefficient of +1 (although it never appears in the map) indicates that a pixel's time course perfectly matches the input signal while a coefficient of -1 indicates that pixel's time course perfectly matches the opposite of the input signal (dotted blue line). At 50 μm the correlation map (color-coded image) shows that the correlation is weak and more dispersed within the ROI in area 3b. At the highest amplitude, however, there is a pronounced and well-defined positive correlation within the ROI that is more evenly distributed throughout the ROI. A large region of negatively correlated activity (corresponding to strong below background activity) surrounds the ROI in the high-amplitude map.

**Figure 8:**
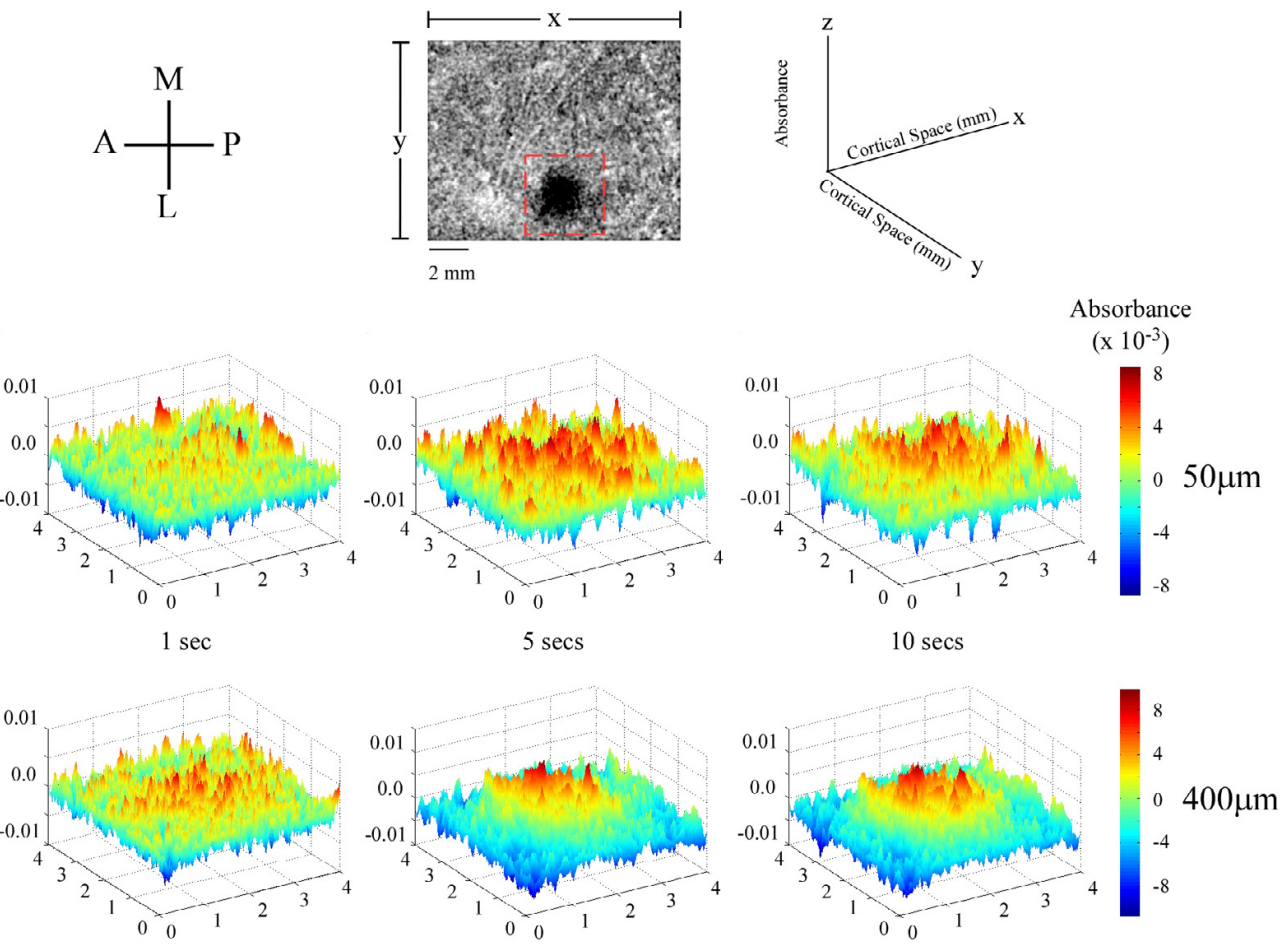
Spatial plots of activity evoked by low- (50 μm) and high-amplitude (400 μm) stimulation. The schematic at the top-left indicates the anatomical orientation of the cortex: A-anterior, M-medial, P-posterior, L-lateral. Absorbances were measured within the boxel shown in the top-middle panel. The schematic (top-right) shows how each frame is spatially represented with respect to the boxel of interest. Both stimulus amplitudes are mapped 1, 5 and 10 seconds after stimulus onset. Absorbance values at each pixel (x, y location) are represented two fold: both by their height along the z-axis, and by their color.

To examine the spatial dynamics of the SI response in more detail we examined the patterns of activity generated by low- and high-amplitude stimulation in a 16 mm^2 ^(4 × 4) area centered around the ROI. Figure 8 demonstrates the patterns of activity evoked at three time intervals during the delivery of the stimulus. The 3D surface plots show activity measured within the boxel indicated by the dashed box in the image at the top. In each 3D plot absorbance is plotted in two-dimensional space and is indicated by two measures: height of the peak along the z-axis (as shown in the schematic at the top right), and the color (indicated by the color bar to the right of each row of 3D plots). These data make it apparent that after a short period of stimulation (1 sec) the activity pattern is very similar for the different amplitudes. That is, at this early time interval both patterns are diffuse and occupy much of the ROI. However as stimulus duration increases, the pattern of increased absorbance evoked by high-amplitude stimulation tends to become restricted to the center of the ROI and within this region becomes homogeneous. Standard deviation was used to measure the variability within the ROI at low and high amplitudes. At 10s after stimulus onset, standard deviations for low- and high-amplitude surface plots were 0.1415 and 0.1166 respectively. Average standard deviation across all sets of maps (n = 5) differs very little from these values (0.1448 at low amplitude vs. 0.1201 at high amplitude). These differences suggest that within the ROI evoked absorbance levels are more homogeneous in response to high- vs low-amplitude stimulation. In addition, at 5 and 10s after onset of high-amplitude (but not low-amplitude) stimulation the territory surrounding the ROI becomes dominated by below-background changes in absorbance.

## Discussion

This study evaluated the global SI response to different amplitudes of flutter stimulation by imaging the optical intrinsic signal (OIS). The OIS indirectly reflects both cortical neuronal spike discharge activity and the local, subthreshold changes in neuronal membrane potential evoked by sensory stimulation [14-16]. As a result, the observed tendency for absorbance in the same localized region of area 3b to increase with increasing stimulus amplitude (Figures 3 &4) most likely is due to the amplitude-dependence of the average firing rate of neurons in the same region [17]. An increase in SI absorbance in response to an increase in stimulus intensity has been reported previously by Chen et al [13]. The observed increase we observed in the stimulus-evoked SI OIS that accompanies increases in stimulus amplitude is well described by a linear function.

One important distinction between previous work done using the OIS and the present study is our use of near-infrared wavelengths for illumination during acquisition of the OIS. The OIS obtained using infrared light has been shown to be highly correlated with light scattering effects that accompany astrocyte swelling subsequent to the clearance of extracellular K^+ ^and neurotransmitter [14,15,18] and local increases in blood volume [19,20]. Although the OIS at this wavelength may be influenced by changes in hemoglobin concentration and oxygenation, it is likely that contributions from these factors are small when compared with light scattering effects[20]. Additionally, OIS imaging using near-infrared illumination not only minimizes the contributions of artifacts introduced by changes in the vasculature (which can dominate the OIS at lower wavelengths) [19] but the time course of the OIS detected at shorter wavelengths (600 nm) is markedly different (shorter) than the protracted OIS observed in this study [13,15,19].

Previous studies conducted by this laboratory have reported that the SI optical response evoked by an extended period (>1 sec) of flutter stimulation not only consists of an increase in absorbance in the region that receives its input from the skin site that was stimulated, but also decreases in absorbance (frequently to levels well below-background) that occur in the surrounding cortex [21]. The present study demonstrates that the below-background component of the SI optical response to flutter stimulation is particularly evident at large stimulus amplitudes (figures 5, 7 &8). However, unlike the increase in absorbance evoked by flutter, the relationship between the magnitude of the stimulus-evoked decrease in absorbance and stimulus amplitude is not satisfactorily described by a linear function. Indeed, the results shown in figure 6 suggest that this component of the optical response to skin flutter is either absent or extremely small at small stimulus amplitudes, and remains maximal or near-maximal across a wide range of suprathreshold stimulus amplitudes. Interestingly, this stimulus-evoked decrease in absorbance (in the surround) appears later in time than the increases in absorbance (within the ROI) and as amplitude is increased it tends to develop earlier after stimulus onset.

The correlation maps shown in figure 7 provide a comprehensive overview of the time course of absorbance at each location in the image. The optical signal at each pixel is cross-correlated with a known input signal. In this case, it is the average absorbance measured within the ROI. The assigned coefficient of determination indicates the degree of similarity between a pixel's time course of absorbance and the input signal. Therefore pixels with a large positive correlation undergo increases in absorbance very similar to the input signal, while pixels with a large negative correlation undergo a decrease in absorbance which more closely resembles the opposite (negative) of the input signal. Figure 8 suggests that at high amplitudes of stimulation the ROI in SI becomes more homogeneously activated with longer stimulus duration. Some evidence for this is indicated by the large discrepancy (between low- and high-amplitude surface plots) in the standard deviations measured within the ROI. Further studies are required to investigate absorbance distribution and patterning within the ROI.

Examination of spatial histograms (figure 2) and the maps in figures 5 &7 also reveal that the size of the SI region that undergoes an increase in absorbance does not increase with increasing stimulus amplitude, but rather remains relatively constant. Regardless of stimulus amplitude, the activated cortical region appears circular in shape and occupies an area approximately 2 mm in diameter (figures 4 &6). Within the ROI average absorbance increases progressively with increasing stimulus duration. The dimensions of the SI region activated by flutter stimulation observed in this study contrast sharply with results presented in previous studies which demonstrated activation within a 1 mm region in diameter [13,22]. One possible explanation for this discrepancy is the level and type of general anesthesia used in the different studies. Previous studies have reported that anesthetics (e.g. ketamine) which block NMDA receptors or enhance GABA_A _receptor mediated inhibition (barbiturates), significantly reduce the dimensions of the receptive field of individual SI neurons; actions that would reduce the size of the responding SI neuronal population [23]. Chen et al. previously reported similar (~2 mm) sized regions of activation in response to flutter stimulation of the digit tips in squirrel monkey anesthetized with isofluorane, as well as showing in the same report that use of pentothal anesthetic confined the response to a much smaller region (~1 mm) [24].

It has been suggested that the amplitude of skin flutter stimulation is coded by both the number of activated SI neurons as well as by their level of spike discharge activity [1]. This suggestion is based largely in part on the fact that larger-amplitude stimuli, through transduction of the laterally-transmitted mechanical wave produced by sinusoidal skin displacement, recruit larger numbers of RA afferents and therefore lead to a spatially more widely distributed pattern of afferent input to SI cortex. Combined metabolic tracer and neurophysiological studies have shown that the initial response to a repetitive tactile stimulus occupies an extremely large cortical territory. As the repetitive mechanical stimulation is continued, however, the response is quickly sculpted by cortical inhibitory mechanisms, leading to an activity pattern that becomes confined to a relatively restricted region in SI [25-27]. The results obtained in the present study and results previously reported by other researchers, lead us to suggest that stimulus amplitude contributes importantly to the shaping (via lateral inhibitory mechanisms) of the SI response to protracted skin flutter.

## Conclusion

This study investigated the SI response to flutter stimulation of the skin using the OIS. An increase of the amplitude of the flutter stimulus was associated with an increase in absorbance within the region of SI cortex that receives its input from the stimulated skin field. The relationship between the maximal change in absorbance and stimulus amplitude is well characterized by a linear function within the range of amplitudes studied. Measurement of the spatial extent of the activated SI region showed that higher amplitudes of stimulation do not produce a more extensive region of SI activation. Instead, as amplitude is increased, while average peak absorbance within the same ~2 mm diameter SI region increases with amplitude of stimulation, the region of surrounding cortex undergoes a prominent decrease (frequently to levels well below background) in absorbance. Further studies are required to establish the relationship between the effect of different amplitudes of skin flutter stimulation on SI absorbance and SI neuroelectrical activity.

## Methods

### Subjects & preparation

All methods and procedures are consistent with USPHS policies and guidelines on animal care and welfare in biomedical research. They were reviewed and approved by an institutional committee prior to initiation of the experiments. Experiments were conducted in 10 squirrel monkeys. Following induction of anesthesia with 4% halothane in a 50/50 mix of nitrous oxide (N_2_O) and oxygen, the trachea was intubated. A veterinary anesthesia machine (Forreger Compac-75) provided an anesthetic gas mix whose composition could be adjusted (typically 1.5–3.0% halothane in 50/50 N_2_O/oxygen) to maintain a stable level of surgical anesthesia. Methylprednisolone sodium succinate (20 mg/kg) and gentamicin sulfate (2.5 mg/kg) were injected intramuscularly to lessen the probability of halothane-induced cerebral edema and prevent bacterial septicemia, respectively. Placement of a valved catheter into a superficial hindlimb vein enabled administration of 5%glucose, 0.9%NaCl, and drugs.

A 1.5 cm opening was trephined in the skull overlying SI cortex. A recording chamber (25 mm i.d.) was placed over the opening and cemented to the skull with dental acrylic. Wound margins were infiltrated with local anesthetic, closed with sutures and bandaged, and the dura overlying SI was resected. After the completion of all surgical procedures subjects were immobilized with norcuron (loading dose 0.25–0.5 mg/kg,i.v.; maintenance dose 0.025–0.05 mg/kg/hr). From this point on, the animal was ventilated with a 50/50 mix of N_2_O and oxygen and the concentration of halothane was adjusted (typically between 0.5 and 1.0%) to maintain heart rate, blood pressure, and the EEG at values consistent with general anesthesia. Rate and depth of ventilation were adjusted to maintain end-tidal CO2 between 3.0 and 4.5%. Under these experimental/anesthetic conditions both SI neuron spontaneous and stimulus-evoked spike discharge activity patterns are highly reproducible over even prolonged (>1 hr) time periods.

### OIS imaging and stimulus protocol

After obtaining a photograph of the exposed cortical surface, the recording chamber was filled with artificial cerebrospinal fluid, and hydraulically sealed using a clear glass plate. In each of five experiments, the OIS evoked in SI by cutaneous flutter stimuli on the thenar region of the forelimb was recorded. The flutter stimulus was delivered at 50, 100, 200 and 400 μm and was interleaved in order to prevent conditioning of the response. The imaging system consisted of a computer-interfaced CCD camera (Quantix 540 from Roper Scentific), light source, guide and filters required for near-infrared (833 nm) illumination of the cortical surface, a focusing device, and a recording chamber capped by an optical window (for additional methodological details see Tommerdahl et al, 1999a,b). Images of the exposed anterior parietal and surrounding cortical surface were acquired 200 ms before stimulus onset ("reference images") and continuously thereafter for 22 s after stimulus onset ("post-stimulus images") at a rate of one image every 0.9–1.4 s. Exposure time was 200 ms. Difference images were generated by subtracting each pre-stimulus image from its corresponding post-stimulus image. Averaged difference images typically show regions of both increased light absorption (decreased reflectance) and decreased light absorption (increased reflectance) which are believed widely (e.g., Grinvald 1985; Grinvald et al. 1991a,b) to be accompanied by increases and decreases in neuronal activation, respectively.

Although onset of the OIS is delayed at longer wavelengths [19,20], use of near-infrared illumination minimizes the contributions to OIS images of the changes in blood flow and flow/volume that normally accompany cortical neuronal activation, and thus may be more useful in the description of spatial characteristics of the signal [20]. All images were examined prior to their inclusion for analysis. Images containing random high amplitude noise were excluded, and the remaining trials (typically ~20) were averaged to improve the signal to noise ratio. OIS images were analyzed using custom routines written in Matlab.

### Correlation methodology

Correlation maps were constructed for comparison of spatial characteristics of the OIS response. This method of analysis has been previously described in detail [28]. Briefly, maps were constructed by choosing a reference region within the imaged field and computing the intensity correlation r_ij _between the reflectance value of each pixel (*i*, *j*) and the average reflectance value within the reference region over the time from stimulus onset to stimulus offset. The region selected as the reference was defined by a boxel (π mm^2 ^area) centered on the region of interest (ROI). Each pixel (*i*, *j*) on the correlation map is represented by a correlation coefficient *r_ij _*(-1 <*r *< 1; - 1 indicates negative correlation; + 1 indicates positive. The statistical significance of each of the correlations was tested with the standard t-test.

### Histological procedures/identification of cytoarchitectural boundaries

At the conclusion of the experiment, the imaged cortical region was removed immediately following intracardial perfusion with saline and fixative. The region then was blocked, postfixed, cryoprotected, frozen, sectioned serially at 30 μm, and the sections stained with cresyl fast violet. The boundaries between adjacent cytoarchitectonic areas were identified by scanning individual sagittal sections separated by no more than 300 mm and were plotted at high resolution using a microscope with a drawing tube attachment. The resulting plots then were used to reconstruct a two-dimensional surface map of the cytoarchitectonic boundaries within the region studied with optical and neurophysiological recording methods. The locations of microelectrode tracks and electrolytic lesions evident in the histological sections were projected radially to the pial surface and transferred to the map of cytoarchitectonic boundaries reconstructed from the same sections. As the final step, the cytoarchitectonic boundaries (along with the locations of microelectrode tracks and lesions whenever present) identified in each brain were mapped onto the images of the stimulus-evoked intrinsic signal obtained from the same subject, using fiducial points (made by postmortem applications of india ink or needle stabs) as well as morphological landmarks (e.g., blood vessels and sulci evident both in the optical images and in histological sections). Locations of cytoarchitectonic boundaries were identified using established criteria [29-31].

## Authors' contributions

SS participated in acquisition of optical data, performed analysis of the data and drafted the manuscript. VT and JC assisted in the data collection and the analysis of the optical imaging data. OF and BW participated in the design of the study, the conduct of the experiments and the drafting of the manuscript. MT participated in the design, conduct, and analysis of the experiments, and in the manuscript preparation.
